# A country report: impact of COVID-19 lockdowns on involuntary psychiatric treatment in Austria

**DOI:** 10.1192/bjo.2023.610

**Published:** 2023-12-12

**Authors:** Matthäus Fellinger, Thomas Waldhör, Benjamin Vyssoki, Michaela Amering, Lisa Leutgeb, Andreas Gschaider, Bernhard Rappert, Daniel König, Gernot Fugger, Philipp Knasmüller, Andrea Gmeiner

**Affiliations:** Clinical Division of Social Psychiatry, Department of Psychiatry and Psychotherapy, Medical University of Vienna, Austria; Centre for Public Health, Department of Epidemiology, Medical University of Vienna, Austria; Clinic Floridsdorf, Department of General Psychiatry, Vienna Healthcare Group, Vienna, Austria; VertretungsNetz, Vienna, Austria; Clinical Division of General Psychiatry, Department of Psychiatry and Psychotherapy, Medical University of Vienna, Austria; Clinic Landstraße, Department of General Psychiatry, Vienna Healthcare Group, Vienna, Austria

**Keywords:** Coercive measures, involuntary admission, mental disorders, lockdowns, COVID-19

## Abstract

**Background:**

Coercive measures such as involuntary psychiatric admission are considered a last resort in the treatment of people with psychiatric disorders. So far, numerous factors have been identified that influence their use. However, the link between a pandemic – in particular, restrictions such as lockdowns – and the use of involuntary psychiatric admission is unclear.

**Aim:**

To examine the association between COVID-19 lockdowns and involuntary psychiatric admissions in Austria.

**Method:**

This retrospective exploratory study assessed all involuntary psychiatric admissions and use of mechanical restraint in Austria, except for the federal state of Vorarlberg, between 1 January 2018 and 31 December 2020. Descriptive statistics and regression models were used.

**Results:**

During the 3-year study period, 40 012 individuals (45.9% females, mean age 51.3 years) had 66 124 involuntary psychiatric admissions for an average of 10.9 days. Mechanical restraint was used during 33.9% of these admissions. In weeks of nationwide COVID-19 lockdowns (2020 *v.* 2018/2019), involuntary psychiatric admissions were significantly fewer (odds ratio = 0.93, *P* = 0.0001) but longer (11.6 (s.d.: 16) *v.* 10.9 (s.d.: 15.8) days). The likelihood of involuntary admission during lockdowns was associated with year (2020 *v.* 2018–2019; adjusted odds ratio = 0.92; *P* = 0.0002) but not with sex (*P* = 0.814), age (*P* = 0.310), use of mechanical restraint (*P* = 0.653) or type of ward (*P* = 0.843).

**Conclusions:**

Restrictions such as lockdowns affect coercive measures and resulted in fewer but longer involuntary psychiatric admissions during weeks of lockdown in Austria. These results strengthen previous findings that showed the dependence of coercive measures on external factors, highlighting the need to further clarify causality and desired prevention effects when using coercive measures.

Coercive measures such as involuntary psychiatric admission (IPA) and the use of mechanical restraint (any freedom-restricting devices including bed rails, movement-restricting blankets or belts) are considered a last resort in the treatment of people with psychiatric disorders. Recently, their use and disadvantages have been discussed widely and intensively, and their prevention, including the implementation of alternatives, has come more into focus.^1–5^ In general, mental health legislation and its practical implementation vary greatly between countries, as do rates of coercive measures between but also within countries, cities or hospitals with similar or identical legal frameworks.^6–9^ Although numerous factors have been identified that influence the use of coercive measures, such as the number of hospital beds per region, medical resources including the patient–staff ratio, staff training and attitudes, and architecture, many questions remain unanswered.^7,8,10–13^ These include how a pandemic and preventive restrictions such as lockdowns affect not only general mental healthcare but also the use of coercive measures. The global outbreak of the COVID-19 pandemic in early 2020, which led to one of the greatest global social, economic and healthcare challenges in recent decades and has jeopardised the regular support and care of people with mental health conditions, has made it possible to pursue this question. Although initial studies on the one hand showed a reduction in aggressive incidents and a concomitant decrease in the use of coercive measures since the onset of the pandemic compared with the period before in a Canadian hospital,^14^ others in Germany observed a decrease in voluntary admissions but an increase in the rates and the duration of coercive measures per case over the course of 2020 compared with 2019.^15–17^ As published studies have so far only compared annual periods before and during COVID-19 (e.g. comparing 2020 with the pre-pandemic year 2019) without taking into account the restrictions in place, the aim of the current study was to examine the association of COVID-19 restrictions such as lockdown periods with IPAs in an Austrian sample.

## Method

### Data source

This nationwide retrospective exploratory study used data from the national patient advocacy VertretungsNetz. In Austria, all psychiatric departments except those in the second-smallest federal state, Vorarlberg, are obliged to report all coercive measures to the VertretungsNetz. Participants’ consent could not be obtained owing to the retrospective design of the study and the pseudonymised nature of the data. A data transfer agreement was agreed between the VertretungsNetz and the Medical University of Vienna and signed by all contractual partners to comply with data protection regulation. Data analysis, data archiving and distribution were done according to the good scientific practice requirements of the Medical University of Vienna and in accordance with the currently applicable requirements and principles of the European General Data Protection Regulation. Furthermore, the authors assert that all procedures contributing to this work comply with the ethical standards of the relevant national and institutional committees on human experimentation and with the Helsinki Declaration of 1975, as revised in 2008. All procedures involving patients’ data were approved by the Ethics Committee of the Medical University of Vienna (EC Nr: 1175/2021).

### Study population

The study included all adults aged 18 years and over who were involuntarily admitted to a psychiatric hospital in Austria (8.9 million inhabitants) between 1 January 2018 and 13 December 2020. However, data for involuntary admissions from the federal state of Vorarlberg (~400 000 inhabitants) were not available, as the national patient advocacy VertretungsNetz is not responsible for data collection in Vorarlberg. Patients being treated in a specific long-term unit for chronic mental disorders, which only exists in one federal state and was closed during the observation period (*n* = 26), were excluded from the study because they were not representative of the available care in Austria. For the analysis, the following variables were used: federal state, type of ward, sex (men/women), year and month of birth, beginning and end of involuntary admission (day), and use of any freedom-restricting devices summarised as mechanical restraint. This last item had to be simplified to ‘present’ or ‘not present’ owing to inconsistent documentation of specific interventions summarising the use of any freedom-restricting devices, beginning from movement-restricting blankets or tables attached to a chair up to mechanical restraint by the use of belts in a bed, as well as other freedom-restricting devices such as protective sleeves to prevent, for example, the removal of essential infusions or self-harm, which are more common in old-age psychiatry.

### Statistical analysis

This was an exploratory study. Accordingly, in the first step, descriptive statistics including means, standard deviations, and/or percentages and quartiles (Q1, Q2 and Q3) were used to describe the continuous characteristics of patients admitted involuntarily in Austria. Numbers of weekly involuntary admissions were compared between 2020 (COVID-19 pandemic) and the previous years (2018 and 2019), with direct comparisons of the weeks of lockdown (weeks 11–20, 46–49 and 51–53). Weekly numbers of involuntary admissions by year as well as the ratio of the corresponding numbers of the two time periods are shown in a figure in order to present the association with lockdown periods graphically. Data were further analysed by chi-squared test for categorical variables and *t*-test for comparison of duration between lockdown groups. Logistic regression analyses were used to test the associations between the prevalence of involuntary admissions within the weeks of lockdown (as the dependent variable) and sex, year (2018–2019 *v.* 2020), use of mechanical restraint and type of department (as independent variables). A linear regression model using the logarithm of duration of admission as the dependent variable was estimated including the same independent variables as mentioned above. The interaction term lockdown period (yes/no) year turned out to be significant; subsequently, the analysis was separately conducted by year (2018–2019 or 2020) to show the associations with sex, lockdown period, use of mechanical restraint and type of department. Generalised estimation equations using SAS procedure GENMOD with correlation structure exchangeable were used to acknowledge that patients may have been admitted more than once during the study period. *P*-values were interpreted in an explorative manner, and correction for multiple tests was omitted. Data management and analysis were done in SAS 9.4.

## Results

### Sample characteristics

During the study period of 3 years (2018–2020), 40 012 individuals were admitted involuntarily a total of 66 124 times. Whereas men had a mean age of 48.7 years and accounted for 54.1% of all involuntary admissions, females were on average 54.4 years old and accounted for 45.9% of all admissions. Men were more often admitted involuntarily at a younger age (up to the age of 45 years), and women had more frequently involuntary admissions from age 75 on. The mean number of admissions per person was 1.58 (95% CI 1.56–1.60) for men and 1.74 (95% CI 1.70–1.78) for women. In 33.9% (*n* = 22 378) of cases, coercive measures with any freedom-restricting devices, as defined in the Methods section, were used. Men (35.9%, *n* = 12 264) faced these measures more frequently than women (31.6%, *n* = 10 114) in total but also in each age group. The detailed sociodemographic characteristics of the study population in regard to the frequency of use of coercive measures can be seen in Table 1.

**Table 1:** Coercive measures in males and females (and total) per age group

Age	Involuntary psychiatric admission (IPA)			Use of mechanical restraint during IPA			
	Men *n* (%)	Women (%)	Total	Men *n* (%)	Women (%)	Total	IPAs involving mechanical restraint (%)
18–30	9215 (56.5)	7100 (43.5)	16 315	3042 (55.6)	2432 (44.4)	5474	33.5
31–45	8443 (52.4)	7653 (47.6)	16 096	2646 (55.6)	2117 (44.4)	4763	29.6
46–60	7578 (49.7)	7669 (50.3)	15 247	2153 (54.9)	1765 (45.1)	3918	25.7
61–75	4058 (50.7)	3940 (49.3)	7998	1582 (58.2)	1137 (41.8)	2719	34.0
76+	4846 (46.3)	5622 (53.7)	10 468	2841 (51.6)	2663 (48.4)	5504	52.6
Total	**34 140 (54.1)**	**31 984 (45.9)**	**66 124**	**12 264 (54.8)**	**10 114 (45.2)**	**22 378**	**33.9**

The mean duration of involuntary admission was 10.9 (95% CI 10.8–11.1) days, and the first, second and third quartiles (Q1, Q2 and Q3) were 2, 5 and 14, respectively. Whereas men had a mean duration of 10.6 (95% CI 10.4–10.7) days (Q1 = 2, Q2 = 5, Q3 = 13), women had been involuntarily admitted for on average 11.3 (95% CI 11.1–11.5) days (Q1 = 3, Q2 = 5, Q3 = 14). Where coercive measures with freedom-restricting devices (mechanical restraint) were used, the mean duration of involuntary admission was 15 days (95% CI 14.7–15.3) (Q1 = 3, Q2 = 8, Q3 = 18), compared with 8.9 (95% CI 8.7–9.0) days (Q1 = 2, Q2 = 4, Q3 = 11) where they were not used. Differences regarding ward type (sex, mechanical restraint and mean duration differences) can be seen in Supplementary Table 3 available at https://doi.org/10.1192/bjo.2023.610.

### Relationship of COVID-19 lockdowns and IPAs

**Fig. 1:**
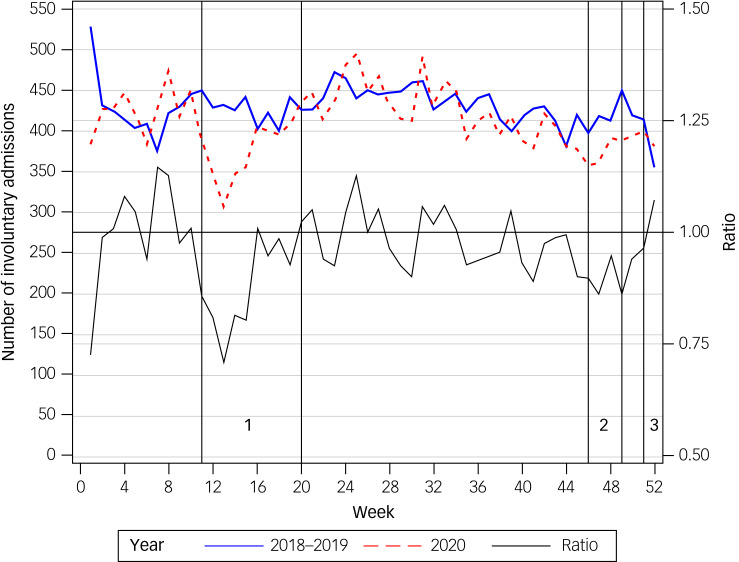
Number and ratio of involuntary admissions per week in 2020 compared with 2018–2019.

The number of IPAs per week in 2020 compared with the two previous years (2018–2019) and the corresponding ratio can be seen in Fig. 1. The strongest decrease in IPAs was during the first COVID-19 lockdown period.

Comparing COVID-19 lockdown periods in 2020 with the same periods in the two previous years (2018 and 2019) with respect to the number of IPAs showed that 22.7% of all IPAs (*n* = 4886) in 2020 took place in lockdown periods, compared with 24.1% in the same time periods in the previous years (*n* = 10 756). Thus, significantly fewer people were admitted involuntarily during periods of COVID-19 lockdowns (*P* = 0.0001), with an odds ratio for being involuntarily admitted during a lockdown period of 0.93 (95% CI 0.96–0.89; adjusted odds ratio = 0.92).

**Fig. 2:**
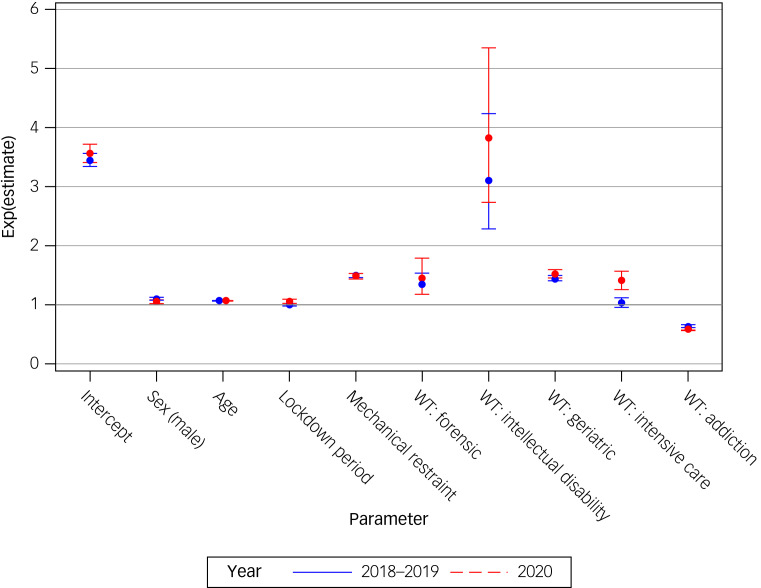
Associations with length of involuntary psychiatric admission per year (2020 *v.* 2018–2019). WT, ward type.

The multivariable logistic regression analysis revealed that the likelihood of being admitted involuntarily during the weeks of lockdown periods was significantly associated with year (2020 *v.* 2018/19; odds ratio = 0.92, *P* = 0.0002) but not with sex (*P* = 0.814), age (*P* = 0.310), mechanical restraint (*P* = 0.653) or type of ward (*P* = 0.843). Additional detailed analysis of generalised estimation equation parameter estimates can be seen in Supplementary Table 4. Furthermore, the duration of IPAs was extended in COVID-19 lockdown periods (2020), with 11.6 (95% CI 11.2–12.1) days compared with 10.9 (95% CI 10.6–11.2) in the previous period (2018/2019). There was a significantly different lockdown effect (interaction term *P* = 0.0033) between years 2020 and 2018–2019 (Supplementary Table 5); therefore, the regression model was calculated separately for years 2018–2019 and 2020. The regression analysis (Table 2), which included all relevant and available parameters described previously, showed that the difference in the duration of IPAs was associated with the lockdown period in 2020 (*P* ≤ 0.0002) but not in the previous years (2018–2019; *P* = 0.530). This difference is shown by the non-overlapping CIs for the years 2020 and 2018–2019 in Table 2. The association between the duration of IPAs with lockdown periods, sex, age, mechanical restraint and type of ward separately for years 2020 *v.* 2018–2019 can be seen in Fig. 2, with general psychiatric care being the reference group for the effect of type of ward.^1^ Thus, it can be observed that the duration of IPAs on wards with a focus on intellectual disability or care of geriatric or forensic patients was increased compared with that of general psychiatric care wards, but without any difference between the observation periods 2018–2019 and 2020. Only the duration of IPAs on intensive care units was increased in 2020 compared with the two previous years.

**Table 2:** Regression model parameters for duration of involuntary admission analysis

Parameter	2018–2019				2020			
	Estimate	95% CI		*P*-value	Estimate	95% CI		*P*-value
Intercept	1.238	1.2066	1.2687	<0.0001	1.271	1.2271	1.3149	<0.0001
Sex (ref. = male)	0.101	0.0787	0.1222	<0.0001	0.0497	0.0198	0.0797	0.0011
Age	0.064	0.0583	0.07	<0.0001	0.0663	0.0581	0.0745	<0.0001
Weeks of lockdown period (ref. = no lockdown)	0.007	−0.0137	0.0267	0.5296	0.0577	0.0272	0.0882	0.0002
Mechanical restraint	0.404	0.3814	0.4272	<0.0001	0.3962	0.3662	0.4263	<0.0001
Ward type								
Forensic	0.299	0.1672	0.4318	<0.0001	0.3747	0.1644	0.585	0.0005
Intellectual disability	1.135	0.8262	1.4444	<0.0001	1.3419	1.0062	1.6776	<0.0001
Geriatric	0.374	0.3416	0.4065	<0.0001	0.4236	0.3774	0.4698	<0.0001
Addiction	−0.443	−0.4803	−0.4049	<0.0001	−0.5291	−0.5752	−0.4831	<0.0001
Intensive care	0.040	−0.0376	0.1169	0.315	0.3428	0.2327	0.453	<0.0001

## Discussion

### Main findings

The current study aimed to determine the impact of COVID-19 lockdowns on IPAs in an Austrian sample, comprising the total population except for the federal state of Vorarlberg (~4% of the population). Our investigation showed that significantly fewer people were involuntarily admitted during the weeks of lockdown compared with the same weeks in 2018 and 2019 (Fig. 1). The reduction in IPAs during lockdown weeks could not be explained by any existing patient or treatment characteristics, apart from the different observation periods in a regression model. Finally, we observed that involuntary admissions were prolonged during lockdown weeks.

### Comparison with findings from other studies

This study was the first to assess the immediate impact of COVID-19 lockdowns on IPAs. As there has been no study to date that would allow a direct comparison of corresponding treatment changes in periods of lockdowns, the results can best be compared with those of studies that examined changes in coercive measures since the beginning of the pandemic. In a representative population, Flammer et al compared treatment characteristics from all voluntary and IPAs in the German federal state of Baden-Wuerttemberg between 2020 and 2019. They found a significantly lower number of voluntary admissions in 2020 compared with the previous year but an increased rate of involuntary treatment and a prolonged duration of the necessity of coercive measures per case.^15^ Similarly, a less representative study observed an increased proportion of urgent or involuntary admissions as well as higher rates of seclusion or mechanical restraint in a private German hospital group in 2020 compared with 2019.^16,17^ Although these two German studies observed increased numbers of involuntary admissions throughout the year 2020, we observed a decrease during periods of lockdown, with the strongest reduction during the first lockdown (calendar weeks 11–20 in 2020; Fig. 1).

### Interpretation of our findings

It has been well documented that the COVID-19 pandemic and the associated restrictions not only changed access to healthcare but also its quality.^18–21^ Mental health services were found to be particularly at risk,^22^ with a World Health Organization survey in 2020 showing that 30% of countries reported partial closure of in-patient mental health services, with rates as high as nearly 70% for community-based services.^23^ Furthermore, available psychiatric and also general healthcare was no longer accessible or provided in the usual way (e.g. reduced availability of general practitioners and out-patient psychiatrists, hospital entry checks by security personnel, use of masks and telemedicine, visit and exit restrictions during in-patient stays). As a result, people were reluctant to seek psychiatric care, resulting in fewer voluntary hospital admissions.^15–17,24^

Particularly during the lockdowns, when access was most restricted, the fewer consultations and clinical assessments and thus fewer identifications of serious illness requiring support and treatment could explain the reduced numbers of IPAs, observed in our study. Reduced social interaction with friends, family and neighbours due to ‘social distancing’ may have had a similar effect, resulting in less identification of support and treatment needs. Furthermore, stricter lockdowns were shown to correlate with less frequent emergency visits among children and adolescents, with a stronger effect if they came from disadvantaged neighbourhoods.^25,26^ As individuals in our study population with severe mental disorders were more likely to live in deprived neighbourhoods, this may also have influenced the results.^27^ In this regard, it should be noted that substance misuse was also limited by changes in drug availability, particularly during the initial preventive COVID-19 restrictions; this may be related to the lower rates of IPA.^28^ As severely intoxicated patients requiring involuntary admission are usually discharged quite quickly, this could result in longer average involuntary treatment length as observed in our study. Thus, the described changes in healthcare and ‘social distance’ were most pronounced during the first lockdown and may explain the low IPA rate during this period.

Another possible explanation of fewer IPAs during lockdowns in our study could be the reduction in psychiatric hospital beds during the COVID-19 pandemic,^23^ as the association of available beds and the number of IPAs has been well described in the literature.^13^ It also seems important to mention the effects of the COVID-19 prevention strategies on the open-door policy of many psychiatric hospitals in Austria,^29^ as a corresponding open-door policy could no longer be maintained, at least temporarily. Open-door policies tend to be associated with lower accommodation rates, at least in Austria.^30,31^

In this context, our observation of prolonged IPAs during the weeks of lockdown could be explained in two ways. On the one hand, it could be an indication of poorer care (less capacity for de-escalation, and fewer visits and therapy offers that would lead to faster improvement). On the other hand, it could be an indication that those who are admitted during a lockdown are more seriously ill. This assumption, including the same observation of prolonged IPAs, is supported by studies comparing mental healthcare characteristics in 2020 with those of the previous year.^15–17^ Flammer et al concluded that the most severely ill patients continued to be cared for, involuntarily if necessary, whereas less severely ill patients either avoided hospital care or were not admitted owing to the very restrictive hospital admission conditions during the pandemic. Furthermore, the authors hypothesise that observed changes might indicate a deterioration in the quality of care owing to the COVID-19 pandemic. These might be a consequence of measures taken to deal with the pandemic, in which the main effort changed from psychiatric care and the prevention of coercive measures to infection prevention.^15^ Thus, when considering involuntary admission as a therapeutic/protective measure, a lower IPA rate during lockdown could also be considered to be a reflection of lower quality of care. The retrospective and exploratory nature of this study and the multiple possibilities for interpretation make it difficult to draw direct conclusions and recommendations for clinical practice. However, studies of the perceptions of care of people with severe mental disorders such as schizophrenia suggest that they experienced significant changes and loss of relevant psychosocial care, particularly during lockdown periods,^32^ highlighting the need to provide the best possible biopsychosocial support to vulnerable groups during pandemic periods and associated restrictions.

### Strengths and limitations

The main strengths of the current study were the individual patient-related and complete population-based data of all Austrian federal states (i.e. 95% of the Austrian population) except for the federal state Vorarlberg. Obviously, a retrospective exploratory study based on service usage data has several limitations. First, it is important to mention that it was not possible to assess causal effects and therefore only associations could be described. Another limitation was the lack of information on diagnoses, psychopharmacological or psychotherapeutic treatments, or specific forms of coercive measures such as seclusion or closed doors. Unfortunately, data could not be linked to any voluntarily received psychiatric care of patients assessed in the study or of all Austrians.

### Future directions

Overall, these results strengthen previous findings that showed a dependence on external factors when using coercive measures; however, they require further clarification and assessment of longer-term consequences. Future studies should assess IPAs and the use of coercive measures in relation to the total number and duration of psychiatric admissions and place those admissions in context of the events aimed to be prevented. Furthermore, international comparisons with countries with different pandemic control policies could provide better information about corresponding changes in mental healthcare and coercive measures.

## Supporting information

Fellinger et al. supplementary material Fellinger et al. supplementary material

## Data Availability

The data that support the findings of this study are available from the corresponding author, T.W., on reasonable request.
