# Supplementary material for: A country report: impact of COVID-19 lockdowns on involuntary psychiatric treatment in Austria
Source: BJPsych Open. 2023 Dec 12;10(1):e9. doi: 10.1192/bjo.2023.610 (PMC10755558; doi:10.1192/bjo.2023.610)
Supplement: Fellinger et al. supplementary material [file S2056472423006105sup001.docx]

# Supplementary Material

*SM Table 3: Involuntary treatment characteristics in regard to type of ward*

|  |  | **Type of ward** | | | | | |
| --- | --- | --- | --- | --- | --- | --- | --- |
|  |  | **Regular** | **Geriatric** | **Addiction** | **Intensive** | **Forensic** | **Intellectual disability** |
| **Total** | N | 49079 | 10163 | 4879 | 1415 | 474 | 114 |
| **Male** | n | 25173 | 5121 | 3222 | 166 | 397 | 61 |
|  | Col % | 51.29 | 50.39 | 66.04 | 11.73 | 83.76 | 53.51 |
| **Female** | n | 23906 | 5042 | 1657 | 1249 | 77 | 53 |
|  | Col % | 48.71 | 49.61 | 33.96 | 88.27 | 16.24 | 46.49 |
| **Mechanical restraint** | n | 14651 | 5271 | 1705 | 334 | 345 | 72 |
|  | Col % | 29.85 | 51.86 | 34.95 | 23.6 | 72.78 | 63.16 |
| **Duration in days** | mean (SD) | 10.2 (14.9) | 17.1 (21.1) | 4.7 (5.4) | 11.5 (16.7) | 14 (17.9) | 34.8 (37.8) |

*SM: Table 4: Regression model parameters for the likelihood of involuntary psychiatric admissions*

| Parameter | Estimate | 95% CL | | p-value |
| --- | --- | --- | --- | --- |
|  |  |  |  |  |
| Intercept | -1.1656 | -1.1656 | -1.1656 | <.0001 |
| Sex (ref=male) | 0.0047 | 0.0047 | 0.0047 | 0.8141 |
| Age | 0.0006 | 0.0006 | 0.0006 | 0.3096 |
| Year (ref=2020) | -0.0786 | -0.0786 | -0.0786 | 0.0002 |
| Mechanical Restraint  (ref=no) | -0.009 | -0.009 | -0.009 | 0.653 |
| Ward type (ref=general psychiatric ward) |  | | | |
| Forensic | -0.128 | -0.3476 | 0.0916 | 0.2533 |
| Intellectual disability | -0.0359 | -0.4604 | 0.3885 | 0.8682 |
| Geriatric | -0.0191 | -0.0802 | 0.0419 | 0.5391 |
| Addiction | -0.0233 | -0.096 | 0.0494 | 0.5302 |
| Intensive care | -0.0065 | -0.1343 | 0.1212 | 0.9205 |

*SM Table 5 Multivariable linear regression analysis of the duration of CIs showing interaction effect of lockdown and year*

| Parameter | Estimate | 95% CL | | p-value |
| --- | --- | --- | --- | --- |
|  |  |  |  |  |
| Intercept | 0.7933 | 0.749 | 0.8375 | <.0001 |
| Sex (ref=male) | 0.0429 | 0.0171 | 0.0687 | 0.0011 |
| Age | 0.0068 | 0.0062 | 0.0075 | <.0001 |
| Period of Lockdown weeks  (ref=no) | 0.0103 | -0.0111 | 0.0317 | 0.3442 |
| Year (ref=2020) | 0.0041 | -0.0164 | 0.0247 | 0.6928 |
| Period of lockdown *Year | 0.0577 | 0.0192 | 0.0962 | 0.0033 |
| Mechanical restraint (ref=no) | 0.3704 | 0.3423 | 0.3985 | <.0001 |
| Ward type (ref=general psychiatric ward) |  |  |  |  |
| Forensic | 0.7409 | 0.6107 | 0.8712 | <.0001 |
| Intellectual disability | 1.7499 | 1.5379 | 1.9619 | <.0001 |
| Geriatric | 0.8535 | 0.8092 | 0.8978 | <.0001 |
| Regular care | 0.4817 | 0.4431 | 0.5203 | <.0001 |
| Intensive care | 0.5903 | 0.5097 | 0.671 | <.0001 |
